# Global Disparities and Trends in Radiotherapy for Early-Stage Glottic Cancer

**DOI:** 10.3390/curroncol33050259

**Published:** 2026-04-29

**Authors:** Issa Mohamad, Shatha Abu Taha, Ahmad Bushehri, Bassem Youssef, Enis Ozyar, Ibrahim Alotain, Ibrahim Abu-Gheida, Mohammed Aldehaim, Carlton Johnny, Layth Mula-Hussain, Majed Alghamdi, Mohamed Shelan, Mohammed Al Dohan, Nadeem Pervez, Olgun Elicin, Saad Alrashidi, Wael El-Sheshtawy, Shoukri Temraz, Zineb Dahbi, Ahmed Abbasi, Abdulrahman Sumaida, Hikmat Abdel-Razeq, Khawla Ammar, Akram Al-Ibraheem, Ali Hosni

**Affiliations:** 1 Department of Radiation Oncology, King Hussein Cancer Center, Amman 11941, Jordan; 2 Department of Radiation Oncology, Kuwait Cancer Control Center, Kuwait City 7065, Kuwait; 3 Department of Radiation Oncology, American University of Beirut Medical Centre, Beirut 11-0236, Lebanon; 4 Department of Radiation Oncology, Acibadem MAA University School of Medicine, Istanbul 34457, Turkey; 5 Department of Radiation Oncology, King Fahad Specialist, Dammam 31444, Saudi Arabia; 6 Department of Radiation Oncology, Burjeel Medical City, Abu Dhabi 92510, United Arab Emirates; 7 Emirates Oncology Society, Dubai 6600, United Arab Emirates; 8 Department of Radiation Oncology, King Faisal Specialist Hospital and Research Center, Riyadh 11211, Saudi Arabia; 9 College of Medicine, Alfaisal University, Riyadh 50927, Saudi Arabia; 10 Department of Radiation Oncology, CancerCare Manitoba, Winnipeg, MB R3E 0V9, Canada; 11 Division of Radiation Oncology, Department of Surgery, Faculty of Medicine, University of British Columbia, Vancouver, BC V5Z 4E6, Canada; 12 Radiation Oncology, Princess Noorah Oncology Center, King Abdulaziz Medical City, Ministry of National Guard Health Affairs-Western Region, Jeddah 11426, Saudi Arabia; 13 College of Medicine, King Saud Bin Abdulaziz University for Health Science, Jeddah 3660, Saudi Arabia; 14 Department of Radiation Oncology, Inselspital, Bern University Hospital and University of Bern, 3010 Bern, Switzerland; 15 Department of Radiation Oncology, King Fahad Medical City, Riyadh 11525, Saudi Arabia; 16 Department of Radiation Oncology, Shifa Tameer- e-Miller University, Islamabad 44000, Pakistan; 17 Department of Clinical Oncology, Al-Azhar University, Cairo 11827, Egypt; 18 Clinical Oncology Department, Mansoura University Hospital, Mansour 35516, Egypt; 19 Department of Radiation Oncology, Mohammed University VI of Health, and Sciences, Casablanca 27182, Morocco; 20 Department of Radiation Oncology, Aga Khan University Hospital, Stadium Road, Karachi 74800, Pakistan; 21 Radiation Oncology Department, Tawam Hospital, Al Ain 15258, United Arab Emirates; 22 Department of Internal Medicine, King Hussein Cancer Center, Amman 11941, Jordan; 23 Office of Scientific Affairs and Research, Survey Unit, King Hussein Cancer Center, Amman 11941, Jordan; 24 Department of Nuclear Medicine, King Hussein Cancer Center, Amman 11941, Jordan; 25 Radiation Medicine Program, Princess Margaret Cancer Centre, University Health Network, Toronto, ON M5G 2M9, Canada; ali.hosni@uhn.ca

**Keywords:** early-stage laryngeal cancer, practice patterns, ipsilateral vocal cord irradiation, whole laryngeal radiotherapy, altered fractionation, hypofractionation

## Abstract

We evaluated global radiotherapy practices in the management of early-stage (AJCC/UICC 8th edition stages I-II) glottic cancer (ESGC). A cross-sectional online survey was conducted in March 2025 across centers worldwide. Data was collected on clinical practices, including staging, CT simulation, target volumes delineation, organs-at-risk contouring, radiotherapy techniques, dose and fractionation schedules, treatment delivery techniques, and image guidance practices. A total of 181 responses were received, primarily from Asia (41.4%) and Europe (24.3%). Most respondents were from non-academic public centers (44.2%), with multidisciplinary team involvement reported by 84.5%. Head and neck CT scan was the most used staging modality (80.1%). Intensity-Modulated Radiation Therapy was the most common planning technique (82.9%). Hypofractionated radiotherapy schedules predominated for T1 (84%) and T2 (72.4%) disease. T1a was typically treated with whole-larynx target volume (72.4%). Use of ipsilateral involved vocal cord irradiation varied by geographical region (*p* = 0.015), being most common in North America (44.8%) and Europe (38.6%). Accelerated fractionation for T2 also differed significantly (*p* < 0.001), with the highest use reported in North America (41.4%). Daily Cone-Beam Computed Tomography was acquired by (58.2%). In total, 70% of respondents expressed interest in the results of a future phase III randomized trial comparing stereotactic body radiation therapy to conventional radiotherapy. Significant global variations in radiotherapy practices for ESGC were observed, likely reflecting disparities in access and differences in institutional protocols. The development and implementation of standardized, evidence-based global guidelines are essential to harmonize care, minimize toxicity, and improve outcomes for patients with ESGC.

## Simple Summary

This study evaluates current global radiotherapy practices among radiation oncologists in the management of early-stage glottic squamous cell carcinoma. The results reveal significant variations in treatment approaches across institutions and countries. These findings highlight the need for international consensus and the development of standardized treatment protocols. Establishing harmonized guidelines may improve treatment quality, promote equitable patient care, reduce radiotherapy-related side effects, and potentially enhance oncologic outcomes for patients with early-stage glottic cancer worldwide.

## 1. Introduction

Laryngeal cancer affects over 188,000 people annually worldwide [1], with early-stage glottic squamous cell carcinoma (ESGC) accounting for a substantial proportion of cases. The standard management for early-stage disease (cTis–T2N0M0) includes transoral endoscopic microsurgery or definitive radiation therapy (RT) [2,3,4], both offering comparable local control (LC) and overall survival (OS) outcomes. Meta-analyses reported 5-year LC rates of 85–95% and OS exceeding 90% for definitive RT [5,6]. RT is often favored for its potential to preserve voice quality, particularly in patients with anterior commissure involvement or relatively large-volume (i.e., bulky) tumors [7]. However, evidence on voice outcomes remains mixed, with some studies showing equivalent results and others suggesting benefits with RT in terms of phonation time and frequency [7,8].

Historically, RT for ESGC was delivered using 2D-conventional or 3D-conformal techniques with opposed lateral fields [9]. More recently, techniques such as vocal cord-only RT (VC-RT) and carotid-sparing Intensity-Modulated Radiation Therapy (IMRT) have been adopted to reduce radiation exposure to surrounding structures while maintaining oncologic efficacy [10,11,12,13]. Retrospective data suggest that VC-RT may offer favorable voice outcomes and comparable LC to whole-larynx RT (WL-RT) [14,15], although prospective data remain limited [10]. Given the concerns regarding laryngeal motions, IMRT with individualized planning target volume margin(s) continues to be explored to minimize RT-associated toxicity [16]. Treatment approaches for ESGC vary widely across institutions and geographical regions [3,4], influenced by local protocols, technological resources, and clinician preference and expertise [16,17]. Oncologic control with voice and functional preservation remains the primary treatment goal [7]. This variation underscores the need for standardized, evidence-based guidelines.

In this study, we conducted a comprehensive global survey to evaluate current RT practices for ESGC. The survey examined differences in staging, treatment planning, target delineation, dose and fractionation schedules, image guidance, and follow-up practices. Understanding these global patterns may help inform future clinical trials and support the development of a global consensus guidelines to harmonize care, reduce toxicity and improve outcomes.

## 2. Materials and Methods

### 2.1. Study Approval

The survey was approved by the Institutional Review Board (IRB) at the King Hussein Cancer Center (IRB number: 25 KHCC 068).

### 2.2. Survey Design

An online survey was developed to assess global variations in RT practices for ESGC. The survey included sections on informed consent, participant demographics, and clinical practices such as staging work-up, multidisciplinary team (MDT) involvement, CT simulation parameters, target volume definition, and organs-at-risk (OAR) contouring, RT techniques, dose and fractionation schedules, treatment delivery techniques, and image guidance practices. The survey was reviewed by ten expert head and neck radiation oncologists for content validity and clarity. A pilot test was performed with 10 participants, and minor revisions were made to improve clarity, based on their feedback prior to final distribution. The full survey instrument is provided as Supplementary Material File S1.

### 2.3. Eligibility and Distribution

Eligible participants were practicing radiation oncologists involved in the management of ESGC. The survey was open from 1–31 March 2025. It was disseminated via two channels: (1) the Middle East Society for Therapeutic Radiation Oncology (MESTRO), which emailed invitations to its full membership, and (2) direct email outreach to corresponding authors of PubMed-indexed publications on laryngeal cancer from January 2010 to July 2024. Participation was voluntary and anonymous; respondents completed the survey after reviewing the informed consent page.

### 2.4. Response Management

Incomplete responses were excluded. Only fully completed surveys were included in the final analysis.

### 2.5. Data Analysis

Data was exported to IBM SPSS Statistics for Windows, Version 27.0 (IBM Corp., Armonk, NY, USA). Descriptive statistics (frequencies, percentages, medians, and ranges) were used to summarize respondent demographics and clinical practices. Proportions of responses for key practice parameters (e.g., staging modalities, simulation techniques, fractionation schedules) were compared across geographic regions using chi-square. A *p*-value of <0.05 was considered statistically significant.

## 3. Results

### 3.1. Respondent Demographics

A total of 181 responses from 54 countries were collected. Of these, 105/1460 were from MESTRO members (response rate: 7%) and 76/1500 from the corresponding authors of the relevant publications (response rate: 5%). Respondents had a mean of 15.1 years of experience (SD ± 8.9). Most were based in Asia (41.4%), Europe (24.3%), or Africa (16.6%). Practice settings included public centers (44.2%), academic centers (38.7%), and private institutions (17.1%) (Supplementary Table S1).

### 3.2. Diagnostic and Staging Approaches

Head and neck CT was the most used modality for locoregional staging (80.1%), followed by MRI (42%). CT chest was the primary tool for distant staging (63%), with ^18^FDG-PET/CT used less frequently (12.2%). Some participants (17.7%) reported no routine staging imaging. Most respondents (84.5%) involved MDTs in treatment decision-making (Supplementary Table S2).

### 3.3. CT Simulation and Treatment Planning

CT simulation typically used 3 mm (45.3%) or 2 mm (38.7%) slice thickness, and Intravenous contrast was used by 50.3% of respondents. Flexible laryngoscopy (FL) informed gross tumor volume (GTV) contouring in 93.9% of responses. Clinical target volume (CTV) delineation practices varied, 51.2% excluded air, 51.8% excluded thyroid cartilage in T1, while 53.7% included it in T2. A 5 mm margin was the most common geometric expansion to create high-dose (68%) and elective-dose (80.7%) CTVs. Isotropic expansions were used to create the planning Target Volume (PTV) in 53% of cases (Table 1).

IMRT was the primary planning technique used by most respondents (82.9%), with 3DCRT used by 40.9%. IMRT use was highest in Europe (95.5%), followed by North America (86.2%) and Asia (76%) (*p* = 0.06). Bolus use for anterior commissure involvement was inconsistent and reported by about one-third of participants. For bulky T2 disease, bilateral elective level II–III nodal irradiation use was common (59.5%), and 26.4% routinely used concurrent chemoradiation (CCRT). Frequently contoured OAR included esophagus, carotid arteries, thyroid, and submandibular glands. Laser therapy was offered as an alternative treatment option to RT by 74.6% of respondents (Table 2).

### 3.4. Fractionation and Dose Regimens

**Figure 1:**
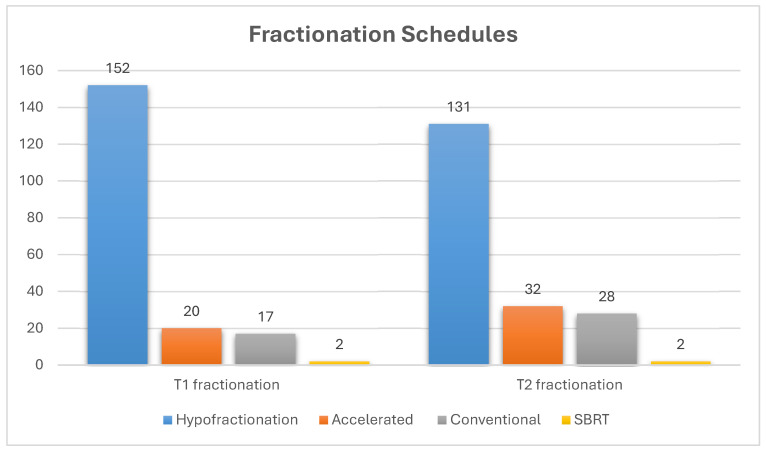
Distribution of fractionation approaches used by respondents (n = 181; multiple responses allowed). The figure shows the number of respondents reporting use of hypofractionation, accelerated fractionation, conventional fractionation, and stereotactic body radiotherapy (SBRT). Multiple responses were permitted. Abbreviations: SBRT: Stereotactic Body Radiation Therapy.

Hypofractionation was preferred for T1 (84.0%) and T2 (72.4%) glottic cancers. The most common regimen for T1 tumors was 63 Gy in 28 fractions (64.6%), and for T2 tumor, 65.25 Gy in 29 fractions (47.5%) (Figure 1). Dose adjustments based on cord involvement were infrequent (16.7%). Conventional fractionation was more common in Africa (47.1%) (*p* = 0.011). WL-RT was used for T1a in (72.4%) of cases; (66.3%) used a single dose level, and (27.6%) used VC-RT. VC-RT use varied significantly by region (*p* = 0.015), being more common in North America (44.8%) and Europe (38.6%). The Danish Head and Neck Study Group (DAHANCA) regimen (70 Gy in 35 fractions, 6 fractions/week) was most used in North America (41.4%) and Asia (13.3%) (*p* < 0.001). (Table 3, Table 4 and Table 5).

### 3.5. Image Guidance and On-Treatment Monitoring

Image guided radiation therapy (IGRT) practices were nearly evenly split between bony (51.4%) and soft-tissue (48.6%) matching (Supplementary Table S3). Daily CBCT was most common (58.2%), Followed by weekly CBCT with daily kV imaging (31.2%). Weekly clinical evaluations during RT were standard (86.7%). Routine FL during treatment was rare (6.6%), while (19.3%) used it as needed.

### 3.6. Follow-Up and Future Directions

Follow-up strategies varied. The most common included FL with neck CT (35.4%), followed by FL with neck/chest CT (24.3%) and FL alone (22.1%). FL with MRI (7.7%) or PET/CT (4.4%) was less common. Overall, FL remained central to surveillance, supplemented variably with imaging. Nearly 70% expressed interest in seeing the results of a future phase III randomized trial comparing stereotactic body radiation therapy (SBRT) to conventional radiotherapy.

## 4. Discussion

### 4.1. Global Variations and Unmet Needs

Management of ESGC varies widely across the globe, driven by disparities in resources, access to technology, and institutional protocols. High-resource centers often employ advanced techniques such as IMRT and VC only-RT to reduce toxicity and preserve function, while many others rely on WL-RT and conventional fractionation due to limited infrastructure and training [18]. Local guidelines, clinician expertise, and available facilities further contribute to inconsistent practices in staging, planning, and follow-up. This variation underscores the need for standardized, evidence-based guidelines and global collaboration to improve care and ensure equitable outcomes.

### 4.2. Staging and Diagnostic Imaging

Head and neck CT was the preferred modality for local staging (80.1%), with MRI used by 42%, aligning with NCCN [19] and Indian [20] guidelines. Despite the low risk of regional and distant metastases in T1–T2 glottic cancers, 63% performed chest CT; while only 12.2% used ^18^FDG-PET/CT, reflecting its limited role in ESGC [21,22]. The NCCN recommends chest CT only for advanced cases, with minimal added value for from PET/CT [19].

### 4.3. Treatment Techniques: IMRT vs. 3DCRT

Both 3DCRT and IMRT were widely used. IMRT allows VC-RT, reduces toxicity and spares carotids, without compromising outcome [23,24,25], though access may be limited in low-resource settings. Among respondents, (82.9%) used IMRT, most commonly in Europe, with a trend toward regional variation (*p* = 0.06).

### 4.4. Vocal Cord-RT vs. Whole Larynx-RT

Global ESGC practices vary, particularly in VC-RT vs. WL-RT use [26], with geography significantly influencing VC-RT adoption in our study (*p* = 0.015). Most clinicians used WL-RT for T1a disease (72.4%). High-resource centers favor VC-RT for its lower toxicity, better voice outcomes, and the ability to avoid elective neck RT [10,27]. WL-RT remains common in lower-income regions due to limited expertise in IGRT, IMRT, and contouring [28]. Training, fractionation preferences, and institutional protocols also play a roles [29]. As resources and training improve, VC-RT use may rise, though disparities reflect broader gaps in RT access.

### 4.5. Anterior Commissure and Bolus Use

Anterior commissure involvement is a negative prognostic factor in ESGC, linked to lower LC [30]. To improve coverage, especially in patients with thin neck anatomy, a bolus is often used [31]. A 5 mm bolus has been shown to improve BED coverage to the target and reduce the negative impact of commissure involvement [32,33]. Still, use is inconsistent, with only 33.7% of respondents applying it routinely.

### 4.6. Fractionation Regimens

This study highlights global variation in ESGC fractionation regimens, particularly between hypofractionated and accelerated RT [34]. Hypofractionation improves LC without added toxicity [4], and accelerated RT (e.g., the DAHANCA with six fractions/week) also enhances control when compared to conventional RT [3]. Most respondents used hypofractionation for T1 (84%) and T2 (72.4%), reflecting its convenience, simple planning, and good outcomes, especially in settings where IMRT is limited [4,35]. In line with the Yamazaki protocol, 66.3% of respondents used a single dose level. Some regions still prefer accelerated RT for better control, less arytenoid edema, or due to institutional protocols and equipment availability [3]. These differences reflect global disparities in resources and infrastructure [36,37].

### 4.7. Target Delineation and Planning Margins

Per ICRU 62 [38], accurate GTV delineation in early-stage glottic cancer requires integrating data from CT/MRI with FL [39,40], as imaging alone may underestimate tumor extent [41]. FL is essential to capture superficial mucosal involvement. Most respondents (93.9%) incorporated FL findings into GTV contours.

Microscopic spread, encompassed by the CTV, typically extends 0–10 mm from the GTV [42,43]. One dose level is often used with hypofractionated RT, while two dose levels are more common with accelerated regimens [41]. Most respondents used 5 mm margins for the high-dose CTV (68.0%) and 10 mm margin for elective CTV (80.7%), aligning with Danish guidelines, which define CTV_1 as GTV + 5 mm and CTV_2 as CTV_1 + 5 mm, receiving therapeutic and prophylactic doses, respectively [44].

CTV contours are anatomically adjusted, excluding air cavities and respecting barriers as the thyroid cartilage per Danish recommendations [41]. PTVs vary by institution [45], but are generally anisotropic, with larger craniocaudal margins for laryngeal motion [46,47]. In our study, 53% used isotropic expansions.

### 4.8. Concurrent Chemoradiation for T2 Disease

The use of CCRT is an option for bulky T2 glottic cancers. “Bulky” is a clinical term used to describe tumors that, while still meeting T2 criteria, are considered large by visual FL and or radiologic assessment. However, the TNM system for laryngeal cancer does not provide a size cutoff or additional criteria for “bulky” within the T2 category [48]. Despite only 26.4% of respondents reporting the used of CCRT, ASCO guidelines endorse it for deeply invasive disease [49]. Retrospective studies show that platinum-based CCRT improves LC, recurrence-free survival, and OS over RT alone, despite higher toxicity [50,51]. A recent trial confirmed better LC and progression free survival (PFS) with CCRT in cases of vocal cord fixation or bulky tumors [52].

### 4.9. Elective Nodal Irradiation

Elective nodal RT remains controversial in ESGC. Unlike supraglottic cancer, nodal spread is rare: almost 0% in T1 and 1.7% in T2. Thus, its benefit is limited and may be outweighed by its associated risks such as long-term carotid toxicity [53,54]. ASCO guidelines also advise against routine elective neck treatment [49]. Despite this, 59.5% of respondents still treated bilateral level II and III nodes for bulky T2 tumors.

### 4.10. Emerging Role of SBRT

SBRT is an emerging option for ESGC, offering precise, short-course RT without requiring elective or contralateral nodal coverage [55,56]. However, its use is limited due to toxicity concerns in the laryngeal area [57] and is best for selected patients with favorable disease. Only 5% of respondents used SBRT, underscoring the need for large trials to confirm its safety, efficacy, and role.

## 5. Limitations

As a cross-sectional, self-reported survey, this study has several limitations. Selection bias may have occurred, as voluntary participation likely favored clinicians with greater interest or expertise in ESGC management, with most respondents from academic or public institutions, limiting generalizability. Non-response bias is also relevant given the low response rate, as non-respondents may differ in practice patterns or resource availability, potentially skewing results. Additionally, self-reported data are subject to recall and reporting biases, including social desirability bias. The survey may not fully capture real-world complexity or local constraints. Despite this, it provides a broad global overview of ESGC management and informs future research and guideline development.

## 6. Conclusions

This survey revealed substantial global variation in the clinical management of ESGC, highlighting a critical need for evidence-based, standardized guidelines. The establishment of consensus-driven protocols is under way to harmonize care, reduce toxicity, and improve outcomes for patients with ESGC worldwide.

## Figures and Tables

**Table 1:** Simulation and target delineation practices among respondents (n = 181).

Variable	Category	Frequency	Percent (%)	95% CI
CT simulation slice thickness	1 mm	20	11	NA
	2 mm	70	38.7	
	3 mm	82	45.3	
	5 mm	9	5	
IV contrast usage for CT simulation	Yes	91	50.3	43–58%
	No	90	49.7	
FL findings used for GTV contouring	Yes	170	93.9	90–97%
	No	11	6.1	
CTVs exclude air	Yes	84	51.2	43–59%
	No	80	48.8	
CTVs exclude thyroid cartilage in T1 disease	Yes	85	51.8	44–60%
	No	79	48.2	
CTVs include inner 1/3 of thyroid cartilage in T2 disease	Yes	88	53.7	46–61%
	No	76	46.3	
Geometric margin expansion from GTV to high-dose CTV (n = 170)	0 mm	8	4.4	NA
	2 mm	2	1.1	
	3 mm	29	16	
	4 mm	1	0.6	
	5 mm	123	68.0	
	>5 mm	18	9.9	
Geometric margin expansion from GTV to elective dose CTV	3 mm	3	1.7	NA
	5 mm	146	80.7	
	10 mm	21	11.6	
Isotropic expansion from CTV to PTV	Yes	96	53.0	46–60%
	No	85	47.0	
CC margin expansion from CTV to PTV	3 mm	54	29.8	NA
	4 mm	1	0.6	
	5 mm	57	31.5	
	10 mm	68	37.6	
	12 mm	1	0.6	
Axial margin expansion from CTV to PTV	3 mm	120	66.3	NA
	4 mm	2	1.1	
	5 mm	53	29.3	
	7 mm	2	1.1	
	10 mm	4	2.2	

Data are presented as number (percentage). Percentages are calculated based on the total number of respondents unless otherwise specified. **Abbreviations**: CC: cranio-caudal; CT: Computed Tomography; IV: Intravenous; FL: Fiberoptic Laryngoscopy; GTV: Gross Tumor Volume; CTV: Clinical Target Volume; PTV: Planning Target Volume.

**Table 2:** Radiation treatment planning and delivery practices (n = 181).

Item	Category	Frequency	Percent (%)
RT technique	3DCRT	74	40.9
	IMRT	150	82.9
	SBRT	9	5.0
	2DCRT	2	1.1
Use of bolus with anterior commissure extension	Yes	61	33.7
	No	58	32.0
	Sometimes	62	34.3
Elective irradiation of bilateral LN II and III for T2 tumors	Yes	97	59.5
	No	29	17.8
	Sometimes	37	22.7
Are bulky T2 tumors treated with CCRT?	Yes	43	26.4
	No	58	35.6
	Sometimes	62	38.0

Data are presented as number (percentage). For items marked with an asterisk, multiple responses were allowed; therefore, percentages may exceed 100%. **Abbreviations**: RT: Radiotherapy; 3DCRT: Three-Dimensional Conformal Radiotherapy; IMRT: Intensity-Modulated Radiotherapy; SBRT: Stereotactic Body Radiotherapy; 2DCRT: Two-Dimensional Conformal Radiotherapy; CCRT: Concurrent Chemoradiotherapy; LN: Lymph Node.

**Table 3:** Fractionation schedules and dose regimens for T1 and T2 ESGC (n = 181).

Item	Category	Frequency	Percent (%)	95% CI
T1 fractionation schedule	Hypofractionation	152	84.0	NA
	Accelerated (6 fx/wk)	20	11.0	
	Conventional	17	9.4	
	SBRT	2	1.1	
T1 Doses	63 Gy/28 fx, 5/wk	117	64.6	NA
	66 Gy/33 fx, 5/wk	21	11.6	
	66 Gy/33 fx, 6/wk	19	10.5	
	51 Gy/20 fx, 5/wk	18	9.9	
	55 Gy/20 fx, 5/wk	13	7.2	
	60 Gy/25 fx, 5/wk	10	5.5	
	50 Gy/15 fx, 5/wk	7	3.9	
	58 Gy/16 fx, 5/wk	3	1.7	
	42.5 Gy/5 fx, 2/wk	3	1.7	
	59.5 Gy/17 fx, 5/wk	1	0.6	
T2 fractionation schedule	Hypofractionation	131	72.4	NA
	Accelerated (6 fx/wk)	32	17.7	
	Conventional	28	15.5	
	SBRT	2	1.1	
T2 Doses	65.25 Gy/29 fx, 5/wk	86	47.5	NA
	70 Gy/35 fx, 5/wk	33	18.2	
	70 Gy/35 fx, 6/wk	28	15.5	
	67.5 Gy/30 fx, 5/wk	18	9.9	
	55 Gy/20 fx, 5/wk	12	6.6	
	60 Gy/25 fx, 5/wk	11	6.1	
	64.8 Gy/27 fx, 5/wk	7	3.9	
	66 Gy/30 fx, 5/wk	5	2.8	
	50 Gy/15 fx, 5/wk	4	2.2	
	66 Gy/33 fx, 6/wk	3	1.7	
	42.5 Gy/5 fx, 2/wk	3	1.7	
	66 Gy/30 fx, 5/wk	2	1.1	
	68 Gy/34 fx, 6/wk	2	1.1	
	69 Gy/30 fx, 5/wk	2	1.1	
Involved cord length affects dose in hypofractionated RT (n = 18)	Yes	3	16.7	−2–36%
	No	15	83.3	
Target volume for T1a tumors	Whole larynx	131	72.4	
	Involved cord only	50	27.6	66–79%
Number of Dose levels	One	120	66.3	59–73%
	Two	61	33.7	

Data are presented as number (percentage). Multiple responses were allowed for fractionation schedules and dose regimens; therefore, percentages may exceed 100%. **Abbreviations**: SBRT: Stereotactic Body Radiotherapy; fx: fractions; wk: week; RT: radiotherapy.

**Table 4:** Comparison of whole larynx versus ipsilateral vocal cord radiotherapy by region (n = 181).

		Whole Larynx (N, %)	Ipsilateral Cord (N, %)	*p* Value	Chi Square	Effect Size Cramer’s V
Region	Africa	23, 76.7%	7, 23.3%	**0.015**	**12.356**	**0.261**
	Asia	63, 84%	12, 16%			
	Australia	2, 66.7%	1, 33.3%			
	Europe	27, 61.4%	17, 38.6%			
	North America	16, 55.2%	13, 44.8%			
	Total	131, 72.4%	50, 27.6%			

Data are presented as number (percentage). *p*-values were calculated using the chi-square test. A *p*-value < 0.05 was considered statistically significant.

**Table 5:** Use of accelerated radiotherapy (70 Gy/35 fractions) versus other schedules by region (n = 181).

		No (N, %)	Yes, (N, %)	Total (N, %)	*p* Value	Chi Square	Effect Size Cramer’s V
Region	Africa	30, 100%	0, 0%	30, 100%	**<0.001**	**21.939**	**0.348**
	Asia	65, 86.7%	10, 13.3%	75, 100%			
	Australia	2, 66.7%	1, 33.3%	3, 100%			
	Europe	39, 88.6%	5, 11.4%	44, 100%			
	North America	17, 58.6%	12, 41.4%	29, 100%			
	Total	153, 84.5%	28, 15.5%	181, 100%			

Data are presented as number (percentage). *p*-values were calculated using the chi-square test. A *p*-value < 0.05 was considered statistically significant.

## Data Availability

Data is available, upon reasonable request, from the corresponding author.

## Supplementary Materials

The following supporting information can be downloaded at: https://www.mdpi.com/article/10.3390/curroncol33050259/s1, File S1: The full survey instrument; Table S1: Institutional Characteristics; Table S2: Diagnostic and Staging Approaches; Table S3: Image Guidance and On-Treatment Monitoring.

## Abbreviations

The following abbreviations are used in this manuscript.

ESGC	Early-stage glottic cancer
LC	Local control
OS	Overall survival
RT	Radiotherapy
VC-RT	Vocal cord-only radiotherapy
IMRT	Intensity-Modulated Radiation Therapy
WL-RT	Whole-larynx radiotherapy
IGRT	Image guided radiation therapy
CBCT	Cone beam computed tomography
SBRT	Stereotactic body radiotherapy
