# Supplementary material for: Global Disparities and Trends in Radiotherapy for Early-Stage Glottic Cancer
Source: Curr Oncol. 2026 Apr 29;33(5):259. doi: 10.3390/curroncol33050259 (PMC13206154; doi:10.3390/curroncol33050259)
Supplement: Supplementary file 1 [file curroncol-33-00259-s001.zip › curroncol-4215245-supplementary.pdf]

# Global Disparities and Trends in Radiotherapy for Early-Stage Glottic Cancer

Thank you for your participation in our study, conducted by Dr. Issa Mohamad, a radiation oncologist at King Hussein Cancer Center in Amman, Jordan. The objectives of this study are to characterize current practices utilized at different centers for early-stage glottic squamous cell carcinoma.

More than one radiation oncologist with experience in head and neck practice per institute is permitted to fill out the survey. The estimated time to complete the survey is 5-10 minutes.

Please note that all data will be anonymized and kept confidential.

If you have any questions, comments, or suggestions, please contact imohamad@khcc.jo.

We sincerely appreciate your valuable contribution to this important research.

**File S1.** The full survey instrument.

* Indicates required question

1. Email address *

_______________________

2. Name of individual filling out the survey *

_______________________

3. Age of individual filling out the survey

*Mark only one oval.*

- ◯ 30-35
- ◯ 35-40
- ◯ 40-45
- ◯ 45-50
- ◯ 50-55
- ◯ 55-60
- ◯ > 60

4. Gender of individual filling out the survey

*Mark only one oval.*

- ◯ Male
- ◯ Female

5. Years of experience in the field *

_______________________________

6. Date of filling out the survey *

_______________________________

*Example: January 7, 2019*

7. Location of cancer center (country) *

_______________________________

8. Name of Radiation Therapy Center (Full Title) *

______________________________

9. Please indicate the type of healthcare facility where you are currently employed *

*Mark only one oval.*

- ( ) Public
- ( ) Private
- ( ) Academic
- ( ) Other: ______________________________

## Clinical Practices

10. What is the imaging modality used at your institution for routine local staging of early- * stage glottic squamous cell carcinoma (SCC)?

*Check all that apply.*

- [ ] Head and neck CT
- [ ] Head and neck MRI

11. What is the imaging modality used at your institution for routine distant staging of * early-stage glottic SCC?

*Check all that apply.*

- [ ] Chest X-ray
- [ ] CT chest
- [ ] PET CT
- [ ] No staging for DM in early-stage glottic cancer

12. Is a multidisciplinary team involved in the management of patients with early-stage glottic SCC at your institution? *

*Mark only one oval.*

- Yes
- No

13. Do you offer a choice between laser treatment and radiotherapy to eligible patients? *

*Mark only one oval.*

- Yes
- No

14. What is the slice thickness used for CT simulation? *

*Mark only one oval.*

- 5mm
- 4mm
- 3mm
- 2mm
- 1mm

15. Do you use IV contrast for CT simulation? *

*Mark only one oval.*

- Yes
- No

16. Do you rely on staging fiberoptic laryngoscopy findings in addition to CT to contour the GTV? *

*Mark only one oval.*

- Yes
- No

17. Do you treat the whole larynx or only the involved cord during irradiation of T1a glottic cancer? *

*Mark only one oval.*

- Whole larynx
- Involved vocal cord

18. How many dose levels do you use for clinical target volume determination? *

*Mark only one oval.*

- One dose level
- Two dose levels (gross and elective dose targets)

19. What is your geometric margin expansion from GTV to create a high dose CTV? *

*Mark only one oval.*

- 0
- 1mm
- 2mm
- 3mm
- 4mm
- 5mm
- >5mm

20. What is your geometric margin expansion from GTV to create an elective dose CTV? *

*Mark only one oval.*

- 10 mm
- 5mm
- 3mm
- Other: ______________________

21. During the process of PTV generation from CTV, is your craniocaudal margin expansion equivalent to axial margin expansion? *

*Mark only one oval.*

- Yes
- No

22. What is your cranio-caudal geometric margin expansion from CTV to create PTV? *

*Mark only one oval.*

- 3mm
- 5mm
- 10mm
- Other: ____________________

23. What is your axial geometric margin expansion from CTV to create PTV? *

*Mark only one oval.*

- 3mm
- 5mm
- 10mm
- Other: ____________________

24. What is the radiation fractionation schedule you use? *

*Check all that apply.*

- [ ] Conventional
- [ ] Hypofractionation
- [ ] Accelerated (DAHANCA)
- [ ] Hyperfractionation
- [ ] SBRT
- [ ] Other: ____________________

25. Please specify the radiation dose you use for **T1** glottic cancer at your institution *

*Check all that apply.*

- [ ] 66 Gy/33 fractions, 6 fractions per week (accelerated)
- [ ] 66 Gy/33 fractions, 5 fractions per week
- [ ] 63 Gy/ 28 fractions, 5 fractions per week
- [ ] 51 Gy/20 fractions, 5 fractions per week
- [ ] 60 Gy/25 fractions, 5 fractions per week
- [ ] 50 Gy/15 fractions, 5 fractions per week
- [ ] 45 Gy/10 fractions, 3 fractions per week
- [ ] 42.5 Gy/5 fractions, 2 fractions per week
- [ ] 36 Gy/3 fractions every other day
- [ ] Other: ______________________

26. Please specify the radiation dose you use for **T2** glottic cancer at your institution *

*Check all that apply.*

- [ ] 70 Gy/35 fractions, 6 fractions per week (accelerated)
- [ ] 70 Gy/35 fractions, 5 fractions per week
- [ ] 67.5 Gy/30 fractions, 5 fractions per week
- [ ] 65.25 Gy/29 fractions, 5 fractions per week
- [ ] 64.8 Gy/27 fractions, 5 fractions per week
- [ ] 60 Gy/25 fractions, 5 fractions per week
- [ ] 79.2 Gy/66 fractions, BID
- [ ] 50 Gy/15 fractions, 5 fractions per week
- [ ] 45 Gy/10 fractions, 3 fractions per week
- [ ] 42.5 Gy/ 5 fractions, 2 fractions per week
- [ ] Other: ______________________

27. Which radiation therapy techniques are utilized at your institution for the treatment of *
early stage glottic cancer?

*Check all that apply.*

☐ 2DCRT
☐ 3DCRT
☐ IMRT
☐ VMAT
☐ SBRT

28. Do you use a bolus for cases with anterior commissure extension? *

*Mark only one oval.*

◯ Yes
◯ No
◯ Sometimes

29. Do you adjust your high and low risk CTVs to **exclude air** in cases of T1 and T2 *
glottic cancer?

*Mark only one oval.*

◯ Yes
◯ No

30. Do you adjust your high and low risk CTVs to **exclude** thyroid cartilage in cases of T1 *
glottic cancer?

*Mark only one oval.*

- Yes
- No

31. Do you adjust your high and low risk CTVs to **include** the inner part (1/3) of the thyroid cartilage in cases of T2 glottic cancer? *

*Mark only one oval.*

- Yes
- No

32. If you use ipsilateral cord irradiation, which organs-at-risk (OARs) do you contour? *

*Check all that apply.*

- [ ] Ipsilateral internal carotid artery only
- [ ] Bilateral internal carotid artery
- [ ] Inferior constrictor muscle
- [ ] Thyroid gland
- [ ] Esophagus
- [ ] Submandibular glands
- [ ] Other: ______________

33. If you use whole larynx irradiation, which OARs do you contour? *

*Check all that apply.*

- [ ] Ipsilateral internal carotid artery only
- [ ] Bilateral internal carotid artery
- [ ] Inferior constrictor muscle
- [ ] Thyroid gland
- [ ] Esophagus
- [ ] Submandibular glands
- [ ] Other: ______________________

34. What type of cone-beam imaging match (IGRT) is performed at your institution? *

*Mark only one oval.*

- ( ) Soft tissue match
- ( ) Bony match

35. What type of image guidance do you use for early-stage laryngeal cancer?

*Mark only one oval.*

- ( ) Daily CBCT
- ( ) Weekly CBCT wit daily KV imaging
- ( ) Daily KV imaging only

36. Do you treat bulky T2 disease with concurrent **chemo**radiotherapy? *

*Mark only one oval.*

- Yes
- No
- Sometimes

37. Do you electively irradiate bilateral cervical lymph node levels II and III in transglottic *
bulky T2 glottic cancer?

*Mark only one oval.*

- Yes
- No
- Sometimes

38. How often do you typically see patients during their radiotherapy course for early-stage *
glottic SCC?

*Mark only one oval.*

- Twice weekly
- Weekly
- Every 2 weeks
- Other: ____________________

39. Do you routinely perform laryngoscopy during the radiotherapy course for early-stage

*

glottic SCC?

*Mark only one oval.*

- Yes, routinely
- Yes, as needed based on symptoms or clinical
- findings No
- Other: ______________________________

40. How do you monitor and follow up with patients who have early-stage laryngeal cancer *

after their treatment?

*Mark only one oval.*

- FOL only
- FOL and CT neck
- FOL with CT neck and

chest FOL with neck

MRI

- FOL with neck MRI and CT

chest FOL with PET/CT

41. If there is a clinical trial that aims to evaluate SBRT with limited volume RT vs

* conventional long course RT, do you think it will be of interest to patients and your institutions to participate in?

*Mark only one oval.*

Y
e
s

N
o

---

**Supplementary Materials:** Supplementary Tables S1–S3 and survey

Supplementary Tables

**Supplementary Table S1.** Institutional Characteristics.

| **Variable** | **Category** | **Frequency** | **(%)** |
|---|---|---|---|
| Region | Africa | 30 | 16.6 |
| | Asia | 75 | 41.4 |
| | Australia | 3 | 1.7 |
| | Europe | 44 | 24.3 |
| | North America | 29 | 16.0 |
| | Total | 181 | 100 |
| Type of healthcare facility | Academic | 70 | 38.7 |
| | Private | 31 | 17.1 |
| | Public | 80 | 44.2 |
| | Total | 181 | 100 |

**Supplementary Table S2.** Diagnostic and Staging Approaches.

| Variable | Category | Frequency | Percent (%) |
|---|---|---|---|
| Imaging used for local staging* | Head and Neck CT | 145 | 80.1 |
| | Head and Neck MRI | 76 | 42 |
| Imaging used for distant staging* | $^{18}$FDG-PET/CT | 22 | 12.2 |
| | CT Chest | 114 | 63 |
| | Chest X-ray | 18 | 9.9 |
| | No DM staging | 32 | 17.7 |
| MDT involvement | Yes | 153 | 84.5 |
| | No | 28 | 15.5 |

**Abbreviations**: PET; Positron Emission Tomography, CT; Computed Tomography, MRI; Magnetic Resonance Imaging, DM; Distant Metastasis, MDT; Multidisciplinary Team

**Supplementary Table S3.** Image Guidance and On-Treatment Monitoring.

| Item | Category | Frequency | Percent (%) |
|---|---|---|---|
| Type of IGRT | Bony match | 93 | 51.4 |
| | Soft tissue match | 88 | 48.6 |
| IGRT imaging technologies | Daily CBCT | 82 | 58.2 |
| | Weekly CBCT + daily KV | 44 | 31.2 |
| | Daily KV only | 15 | 10.6 |
| Frequency of patient evaluations during RT | Weekly | 157 | 86.7 |
| | Twice weekly | 12 | 6.6 |
| | Every 2 weeks | 10 | 5.5 |
| | As needed | 2 | 1.1 |
| Routine laryngoscopy during RT | No | 134 | 74.0 |
| | Yes, as needed | 35 | 19.3 |
| | Yes, routinely | 12 | 6.6 |

**Abbreviations:** IGRT: Image-Guided Radiotherapy; CBCT: Cone Beam Computed Tomography; KV: Kilovoltage; RT: Radiotherapy.
